# IL 10 gene -1082 G/A shows an associatoin with rheumatoid arthritis patients of South Indian Population

**DOI:** 10.1186/1755-8166-7-S1-P114

**Published:** 2014-01-21

**Authors:** EK Krishna Priya, Moinak Banerjee, S Rajesh, K Sasikala

**Affiliations:** 1 Department of Zoology, Bharathiar University, Coimbatore; 2 Rajiv Gandhi Center for Biotechnology, Trivandrum, India; 3 Department of Rheumatology, KIMS Hospital, Trivandrum, India

## Background

Rheumatoid arthritis (RA) is a systemic, chronic and inflammatory joint disease of unknown etiology with genetic predisposition. This disease is three times more frequent in women than men and is strongly associated with the circulating auto-antibodies like rheumatoid factor (RF) and anti cyclic peptide antibody (ACPA). The cytokine network dysfunction plays a vital role in the pathogenesis of rheumatoid arthritis. The purpose of the study was to understand the autoantibody specificities and its association with Disease activity score Interleukin 10(-819 T/C, -592 A/C & -1082 G/A) promoter polymorphisms in rheumatoid arthritis patients of South Indian population.

## Materials and methods

According to ACR criteria 1987, 168 rheumatoid arthritis patients and 244 controls groups were selected as study population. Genotyping of IL-10 promoter regions -819T/C and -592 A/C were performed by PCR- Direct sequencing and -1082 G/A promoter polymorphism by PCR- RFLP method.

## Results

The results show that -1082 G/A promoter region shows a significant association with rheumatoid arthritis patients in South Indian population (genotypic p = 2 x 10^-4^ & allelic p = 2 x 10^-5^). The disease activity score were positively correlated with rheumatoid factor of RA patients (p = 4 x 10^-2^).

## Conclusions

The study concludes that the allele G of -1082 promoter region predisposes susceptibility to Rheumatoid Arthritis. We also found a strong association of positive rheumatoid factor with the disease phenotype in RA patients.

